# Osteocalcin reduces fat accumulation and inflammatory reaction by inhibiting ROS-JNK signal pathway in chicken embryonic hepatocytes

**DOI:** 10.1016/j.psj.2022.102026

**Published:** 2022-06-24

**Authors:** M. Zhang, W.J. Tu, Q. Zhang, X.L. Wu, X.Y. Zou, S. Jiang

**Affiliations:** ⁎ Joint International Research Laboratory of Animal Health and Animal Food Safety, College of Veterinary Medicine, Southwest University, Chongqing, 400715, China; † Immunology Research Center, Medical Research Institute, Southwest University, Chongqing, 402460, China

**Keywords:** osteocalcin, fatty liver hemorrhagic syndrome, chicken embryonic hepatocyte, ROS-JNK, fat accumulation, inflammatory reaction

## Abstract

Osteocalcin (**OCN**) has a function in preventing fatty liver hemorrhagic syndrome (**FLHS**) in poultry. The aim of this study was to investigate the effects of OCN on fat emulsion stimulated chicken embryonic hepatocytes and related signaling pathways. The primary chicken embryonic hepatocytes were isolated from the incubated 15-day (**E15**) pathogen free eggs and cultured with dulbecco's modified eagle medium (**DMEM**). After the hepatocyte density reached 80%, the cells were divided into 5 groups: control group (**CONT**), fat emulsion group (**FE**, 10% FE, v/v), FE with ucOCN at 1 ng/mL (FE-LOCN), 3 ng/mL (FE-MOCN), and 9 ng/mL (FE-HOCN). In addition, 2 mM N-Acetyl-L-cysteine (**NAC**) a reactive oxygen species (**ROS**) scavenger, and 5 μM SP600125, a Jun N-terminal kinase (**JNK**) inhibitor, were added separately in to the DMEM with 10% FE to test effects of FE on the function of ROS-JNK signal pathway. The number of hepatocytes, cell ultra-microstructure, viability, and apoptosis were detected after 48 h treatment, and the protein expressions and enzyme concentrations were detected after 72 h treatment. The results showed that, compared to the control group, FE increased the triglyceride (**TG**) concentration and lipid droplets (**LDs**) in chicken embryonic hepatocytes (*P* < 0.05), and induced hepatocytic edema with obviously mitochondrial swelling, membrane damage, and cristae rupture. FE also decreased ATP concentration, increased **ROS** concentrations and mitochondrial DNA (**mtDNA**) copy number, promoted inflammatory interleukin-1 (**IL-1**), IL-6, tumor necrosis factor-alpha (**TNF-α**) concentrations and hepatocytic apoptosis rate, and raised phospho-c-Jun N-terminal kinase (**p-JNK**) protein expressions. Compared to the FE group, ucOCN significantly increased hepatocyte viability, reduced hepatocytic TG concentrations and LDs numbers, and alleviated hepatocytic edema and mitochondrial swelling. Furthermore, ucOCN significantly decreased ROS concentrations, increased ATP concentrations, reduced IL-1, IL-6, TNF-α concentrations and hepatocytic apoptosis rate, and inhibited p-JNK protein expressions (*P* < 0.05). NAC had the similar functions of ucOCN reduced the ROS concentration and inhibited the TNF-α protein expression and p-JNK/JNK ration. Similarly, SP600125 reduced p-JNK/JNK protein expression, IL-1, IL-6, TNF-α, and TG concentrations without effects on ROS concentration and hepatocytic apoptosis. These results suggest that ucOCN alleviates FE-induced mitochondrial damage, cellular edema, and apoptosis of hepatocytes. These results reveal that the functions of ucOCN in reducing fat accumulation and inflammatory reaction in chicken embryonic hepatocytes are mostly via inhibiting the ROS-JNK signal pathway.

## INTRODUCTION

Fatty liver hemorrhagic syndrome (**FLHS**) is one of the main metabolic diseases in hens, which is characterized by increased lipid accumulation, fragile liver, rupture bleeding, and sudden death (Rozenboim et al., 2016). Approximately 40% of the hens died due to FLHS, and the data even up to 74% in caged laying hens (Shini et al., 2019). Multiple factors, such as nutrition, environment, hormone, metabolism, and gene, are involved in occurrence of FLSH (Choi et al., 2012; Song et al., 2017; Li et al., 2020b), and nutrition is the most important factor for hens, especially commercial hens fed over-nourishment diet for maintaining high production (Choi et al., 2012). Hens’ FLHS has similar nosogenesis and pathophysiological mechanism as mammal nonalcoholic fatty liver disease (**NAFLD**) (Sánchez-Polo et al., 2015; Hamid et al., 2019), which also inculdes a spectrum of disorders ranging from the simple fatty liver to steatohepatitis, with increasing fibrosis leading to cirrhosis (Leoni, et al., 2018). Simple fatty liver induced by over-nourishment is the first critical step of FLHS. The experiment hens’ FLHS model can be successfully induced by high-fat and high-fat (energy) low-protein diets (Zhu et al., 2020; Qiu et al., 2021; Tan et al., 2021). Moreover, research showed that 97% FLHS hens were obese, which had a large amount of subcutaneous and intracoelomic fat accumulation (Trott et al., 2014). The expression of hepatic de novo lipogenesis genes are increased and fatty acid β-oxidation genes are decreased in the high-energy and low-protein diet-induced FLHS in laying hens (Miao et al., 2021), resulting in great accumulations of free fatty acid, triglyceride (**TG**) and lipid drops (**LDs**) in the FLHS hens’ liver (Lin et al., 2021; Miao et al., 2021). Therefore, preventing simple fatty liver in laying hens is the key step to prevent FLHS.

Osteocalcin (**OCN**) is a 49-amino noncollagenous bone matrix protein produced by osteoblasts and has functions in regulating energy metabolism in an active undercarboxylated form (**ucOCN**) (Lin et al., 2018). Clinical data have showed that both total and ucOCN are inversely associated with liver steatosis, inflammation, ballooning, and fibrosis grades in NAFLD patients (Yilmaz et al., 2011; Du et al., 2015; Xia et al., 2021). ucOCN can prevent NAFLD development in mice by enhancing hepatocytic insulin sensitivity and promoting proliferation and functions of pancreatic β-cells (Zhang et al., 2020), inhibiting hepatic lipid synthesis, promoting lipolysis processes (Zhang et al., 2021), preventing inflammation and fibrosis (Gupte et al., 2014). Similarly, our previous study in chickens has reported that ucOCN alleviates FLHS process through reducing hepatic haemorrhage and fibrosis, and inhibiting insulin resistance, inflammation, and oxidation stress in high-fat diet (**HFD**)-fed aged laying hens (Wu et al., 2021b). However, the effect of ucOCN on the simple fatty liver has not been fully elucidated.

Reactive oxygen species, including superoxide anion radicals (**O_2_^•−^**) and hydrogen peroxide (**H_2_O_2_**), are continuously produced intracellularly as byproducts of energetic metabolism in hepatocytes of NAFLD (Zhao et al., 2020; Dabravolski et al., 2021). Hepatic lipid overload induces the overproduction of oxidants by affecting the mitochondria, peroxisomes, and endoplasmic reticulum. The nonelectron transport chain sources of ROS, especially the β-oxidation of fatty acids, appear to be the major source of ROS in hepatic metabolic disorders (Chen et al., 2020). The dysregulation of liver lipid metabolism in NAFLD mice generates higher levels of ROS (Ma et al., 2016). Palmitic acid induces rat primary hepatocytes simple steatosis associated with excessive ROS production (Moravcová et al., 2015). At high concentrations, ROS causes oxidative modifications to cellular macromolecules (DNA, lipids, proteins, etc.) (Jakubczyk et al., 2020) and leads to activation of the c-Jun N-terminal kinase (**JNK**), consequently inducing liver damage (Schwabe and Brenner, 2006; Li et al., 2020a).

The c-Jun N-terminal kinase is a mitogen-activated protein kinase (**MAPK**) family member that mediates cellular responses to a variety of intra- and extracellular stimulations (Czaja, 2010). The JNK isoforms are encoded by three genes, two of which are JNK1 and JNK2 expressing in all cells including hepatocytes (Weston and Davis, 2002). Investigations have indicated that overactivation of JNK is crucial to NAFLD process (Weston and Davis, 2002; Schattenberg et al., 2006; Yan et al., 2017). JNK mediates NAFLD development by involving in obesity, insulin resistance, lipid accumulation, and liver fibrosis (Czaja, 2010). Inhibition of JNK attenuates insulin resistance in NAFLD rats (Yan et al., 2017). JNK1 null mice have significantly low levels of steatohepatitis after fed the methionine- and choline-deficient (**MCD**) diet (Schattenberg et al., 2006). In addition, therapeutic effects of ucOCN in NAFLD mice may be intervened by activating the nuclear factor like-2 (**Nrf2**) pathway to alleviate oxidative stress and to inhibit the JNK pathway in hepatocytes (Du et al., 2016). Melatonin improves NAFLD by reducing inflammation in HFD-induced obese mice through modulating the MAPK-JNK/P38 signaling pathway (Sun et al., 2016). The ROS-JNK signal pathway participates in the NAFLD process. We spectacled ROS-JNK involves in chicken embryonic hepatocyte steatosis, and ucOCN prevents the chicken embryonic hepatocyte steatosis via regulating the ROS-JNK signal pathway. Therefore, the effect of ucOCN on chicken embryonic hepatocytes steatosis induced by 10% fat emulsion (**FE**) was further investigated.

## MATERIALS AND METHODS

### Primary Chicken Embryonic Hepatocytes Isolation and Culture

Primary hepatocytes were prepared as described before (Arias, 2012). Briefly, the livers were dissected out from ten 15-day (E15) old chicken embryo without specific pathogens (Shandong Haotai Experimental Animal Breeding Co., Shandong, China). The liver samples were cut into small pieces (1 mm × 1 mm) and digested by 0.25% trypsin-EDTA (Gibco, New York, USA) at 37°C for 30 minutes. The hepatocytes were collected by low-speed centrifugation (1000 r/min × 5 min), and further purified by Percoll gradient centrifugation (60 % v/v, Biosharp, Anhui, China). chicken embryonic hepatocytes at 2 × 105 cells/mL were cultured with dulbecco's modified eagle medium (**DMEM**, Gibco, New York, USA) in a humidified atmosphere of 95% air and 5% CO_2_ at 37°C.

### Experimental Design

The hepatocytes were grown in 6-well cell culture plates. After the hepatocytes density reached 80%, the cells were harvested and treated by adding FE with or without ucOCN (Mybiosource, San Diego, CA) into the DMEM for studying the effect of ucOCN on chicken embryonic hepatocytes. There were 5 groups: control group (**CONT**), FE group (**FE**, 10% FE, v/v), FE with ucOCN 1 ng/mL (**FE-LOCN**), 3 ng/mL (**FE-MOCN**), and 9 ng/mL (**FE-HOCN**). SP600125 (a JNK inhibitor, Beyotime, Shanghai, China) and NAC (a ROS scavenger, Beyotime, Shanghai, China) were used for investigating the effects of FE on the function of ROS-JNK signal pathway of the hepatocytes. Based on our pilot study, in which 0.5, 1, and 2 mM NAC and 2.5, 5, and 10 μM SP600125 were used, in this study, 2 mM NAC and 5 μM SP600125 were added with 10% FE at the same time in the cell culture fluid for testing the effects on FE on the function of ROS-JNK signal pathway. The number of hepatocytes, cell ultra-microstructure, viability, and apoptosis were detected after 48 h treatment, and protein expressions and enzyme concentrations were detected after 72 h treatment.

### Cell identification and Viability Assay

Cells were washed with phosphate buffer saline (**PBS**) for 3 times, fixed with 4% paraformaldehyde for 30 min, then stained with Periodic acid-Schiff (**PAS**, Beyotime, Shanghai, China) for 15 min. The cells with the red granules were identified as hepatocytes under light microscope (Leica DM500, Leica microsystems, Wetzlar, Germany). The hepatocytes viability detected by CCK-8 (Biosharp, Hefei, China) method. Hepatocytes were cultured in 96-well plates for 4 h with 10 μL CCK8 per well. Afterwards, Optical density (**OD**) values were measured using a microplate reader (ThermoFisher, Waltham, USA) at the 450 nm wavelength.

### Cell Ultra-Microstructure Analyses

The ultra-microstructure of hepatocytes was observed and analyzed at the Wuhan Servicebio technology CO., LTD (Wuhan, China). The hepatocyte precipitation was collected by centrifugation, re-suspended in the TEM fixation (Servicebio, Wuhan, China) for 120 min, rinsed by 0.1M phosphoric acid buffer (**PB**, pH7.4) for 15 min, then postfixed with 1% osmic acid 0.1M PB for 120 min. Followed centrifuged, the fixed hepatocytes precipitation was dehydrated with serially diluted ethanol, penetrated with acetone and EMBed 812 (1:1, SPI, West Chester, PA), and embedded with EMBed 812, then cut to 60 to 80 nm by ultra-microtome (Leica microsystems, Wetzlar, Germany). The sections were stained by using 2% uranium acetate saturated alcohol solution and 2.6% lead citrate. The specimens were observed under transmission electron microscope (Hitachi, Japan).

### Concentrations of Oxidative Damage Factors of Hepatocytes

Oil red O staining kits (Nanjing Jiancheng Bioengineering Institute, Nanjing, China) were used for light microscopic examination and quantitative analysis according to the manufacturer's instructions. The concentrations of TG, malondialdehyde (**MDA**), glutathione peroxidase (**GSH-Px**), superoxide dismutase (**SOD**), and adenosine triphosphate (**ATP**) in the hepatocytes were measured by the relative assay kits (Nanjing Jiancheng Bioengineering Institute, Nanjing, China) by followed the company's protocols.

Total DNA of hepatocytes were extracted by followed the genomic DNA preparation protocol (Beyotime, Shanghai, China). The primer of ND1 of mitochondrion specific gene (F:5’GAGCCAATCCGACCATCTAC, R:5’GGGACTCAAATAGTCAGGGC) and 18s RNA of nuclear genome (5’GTCTAAGTACACACGGGCGG, R:5’CCTTGGATGTGGTAGCCGTT) were designed using the Primer 5.0, synthesized by the Invitrogen Biotechnology (Shanghai, China). The copy number of ND1 and 18s RNA were analyzed by real-time PCR (BIO-RAD, California, USA), and the relative mitochondrion DNA (**mtDNA**) copy number was calculated according to the ratio of ND1 and 18s RNA (Abu-Amero et al., 2014).

### Cell Apoptosis by Flow Cytometry

Cell apoptosis was detected using the Annexin V-fluorescein isothiocyanate/propidium iodide (**Annexin V-FITC/PI**, BestBio, Shanghai, China) method (Zhang and Liang, 2014). Briefly, the hepatocytes were washed with cold PBS for 3 times, digested with 0.25% trypsin-EDTA, centrifuged, then resuspended with 300 μL Binding Buffer in a 1.5 mL EP tube. Added 5 μL Annexin V-FITC in each tube, gently mixed, then placed in a dark place at room temperature (20–25°C) for 15 min. After added 5 μL PI, the stained hepatocytes were detected by the FITC and PI channels of the flow cytometry (ACEA NovoCyte, Hangzhou, China) within 1 h. Flow cytometry data was analyzed by the NovoExpress software (Beijing Aiqinghai Co., Beijing, China).

ROS activity was detected by using fluorescent probe 2,7-Dichlorodihydrofluorescein diacetate (**DCFH-DA**, BestBio, Shanghai, China). DCFH-DA was diluted with no serum medium to 10 μM. The hepatocytes were collected, counted, and suspended using DCFH-DA solution to 10^7^ cells/mL. The hepatocytes were incubated for 20 min at 37°C, then washed with PBS. DCFH entering the cells is oxidized by ROS to fluorescent DCF, and the density of DCF fluorescence positively correlated with ROS activity was detected through FITC channel of the flow cytometry (ACEA NovoCyte, Hangzhou, China). Flow cytometry data was analyzed by the NovoExpress software (Beijing Aiqinghai Co., Beijing, China).

### Enzyme-Linked Immunosorbent Assay (ELISA)

The levels of interleukin-1 (**IL-1**), IL-6 and tumor necrosis factor-alpha (**TNF-α**) were measured by using relative ELISA kits (Xiamen Huijia Biotechnology Co., Ltd, Fujian, China) which were specific for chickens. Each absorbance value was read by using a microplate reader (ThermoFisher, Waltham, USA).

### Western Blotting

The total proteins of hepatocytes were extracted by radio immunoprecipitation assay (**RIPA**) lysate (ThermoFisher, Waltham, USA) containing both protease inhibitor and phosphatase inhibitor (Beyotime, Shanghai, China). Briefly, the concentrations of protein samples were detected by using Protein Assay Kit (Beyotime, Shanghai, China). A volume of 30 μg proteins from each sample was separated by 10% to 15% SDS-polyacrylamide gels and transferred to polyvinylidene fluoride (**PVDF**). The films were soaked with phosphate buffer saline tween (**PBST**), blocked with 5% skimmed milk powder solution at 4°C for overnight, then incubated with the primary antibodies of TNF-α (26 kDa; Mouse Anti-TNF alpha monoclonal antibody; 1:1000; bsm-33207M; Bioss, Beijing, China), p-JNK (50kDa; Rabbit Anti-phospho-JNK1 (Thr183) polyclonal antibody; 1:1000; bs-17591R; Bioss, Beijing, China), JNK (42 kDa; Rabbit Anti-JNK1+JNK3 polyclonal antibody; 1:1000; bs-20760R; Bioss, Beijing, China), Nrf2 (110kDa; Rabbit Anti-Nrf2 polyclonal antibody; 1:1000; bs-1074R; Bioss, Beijing, China) or glyceraldehyde-3-phosphate dehydrogenase (**GAPDH**, 36 kDa; mouse anti-GAPDH-loading control antibody; 1:5000; ab8226; Bioss, Beijing, China) gently shaken for 2 h at room temperature. The films were further incubated with the HRP-conjugated goat anti-rabbit antibody (1:5000; ab205718; Beyotime, Shanghai, China) or HRP-conjugated goat anti-mouse antibody (1:5000; ab205719; Beyotime, Shanghai, China) for 2 h at room temperature. Finally, SignalFire plus ECL reagent (ThermoFisher, Waltham, USA) was used to show the target bands, then the ImageJ software (version 5.0, BIO-RAD, California, USA) was used to analyze and count the gray value of the bands. The target band was normalized to GAPDH in each lane, and each plate was homogenized by the first hole.

### Statistical Analyses

The data were analyzed by using SPSS 22.0 (IBM Co., New York, USA). A One-way ANOVA was used to analyze the differences among the groups. Data normality was checked. Post hoc multiple comparisons were performed using LSD's test. Values were expressed as mean ± SEM, and *P*-value <0.05 was defined statistically significant.

## RESULTS

### Effects of ucOCN on FE-Induced Chicken Embryonic Hepatocytes Viability and Fat Accumulation

**Figure 1:**
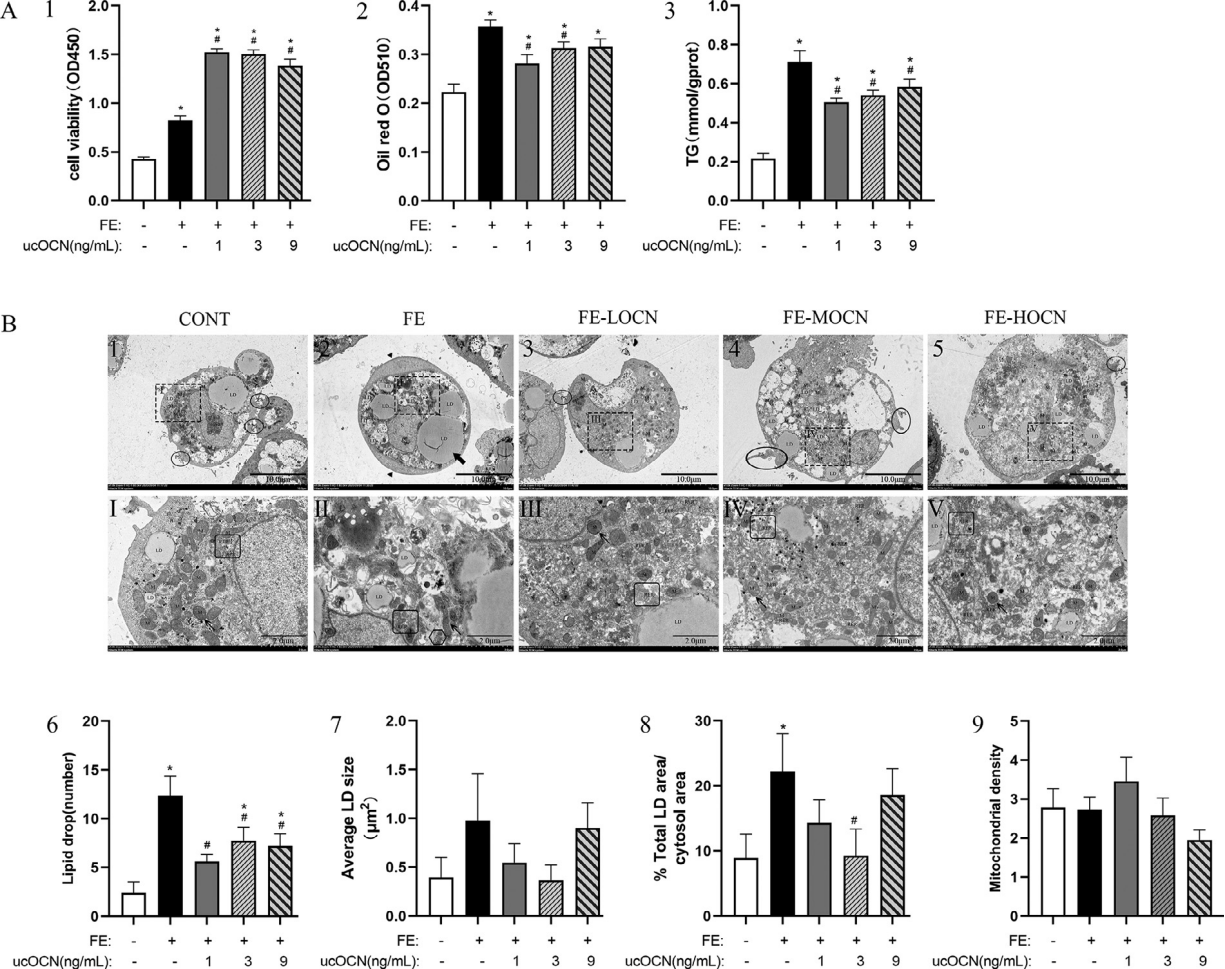
Effects of ucOCN on FE-induced chicken embryonic hepatocytes viability and fat accumulation. A1-3, The cell viability and fat accumulation detected by oil red O and TG. B1-5, Examples of electron micrographs showing hepatocytic ultrastructural features. BⅠ-Ⅴ, High power micrographs of the relative areas outlined by the squares. Bar = 10 or 2 µm, respectively. B6-9, Quantitative analysis of LD and mitochondria density. The data represent Mean ± SEM (n = 6 per group). Differences were determined by one-way ANOVA followed by LSD test. * *P* < 0.05, compared with the control group, # *P* < 0.05 compared with the FE group. Photo markers: ▲: cellular edema; **○**: pseudopodia; **↑**: mitochondrial structure; : rough endoplasmic reticulum structure; ⇧:Golgi hypertrophy; : large LDs; Abbreviations: OCN, osteocalcin; FE, fat emulsion; TG, triglyceride; LD, lipid drop; PS, pseudopodia; N, nucleus; Nu, nucleolus; M, mitochondria; RER, endoplasmic reticulum; GL, glycogen.

The reddish granules in the cytoplasm of cells identified as hepatocytes were isolated and purified for the following analyses. CCK-8 analysis showed that the FE group hepatocytes had higher viability (*P* < 0.05; Figure 1A1) than the cells of CONT, and the different ucOCN concentrations further improved the cell viability compared to the FE group (*P* < 0.05). Oil red O staining and cellular TG content analysis showed that compared to the control cells, FE significantly increased the chicken embryonic hepatocytes fat accumulation (*P* < 0.05; Figure 1A2) and the TG concentrations (*P* < 0.05; Figure 1A3). ucOCN regardless of its concentration decreased the hepatocytes fat and TG accumulation by FE, however, trended to be the lowest in FE-LOCN group.

The transmission electron microscopic image observation indicated that compared with CONT (Figure 1B1, BⅠ), the hepatocytes of the FE group (Figure 1B2, BⅡ) had more damage with cellular edema, higher intracellular electron, cell membrane smooth, pseudopodia disappeared, great mitochondrial swelling with part membranolytic, cracked or decreased of crista, increased dilated or degranulated rough endoplasmic reticulum, and Golgi hypertrophy. Oil red O staining also showed that FE caused hepatocytes ultrastructure damage, resulting in more LDs in hepatocyte plasma than control cells (*P* < 0.05; Figure 1B6), and most of them fused to form large LDs, and the % Total LD area/cytosol area increased significantly (*P* < 0.05; Figure 1B8). ucOCN reduced the LDs quantity to some extent without obvious fusion and 3 ng/mL ucOCN could significantly reduce the % Total LD area/cytosol area (*P* < 0.05).

### Effects of FE and ucOCN on Mitochondrial Function and Oxidative Stress in Chicken Embryonic Hepatocytes

**Figure 2:**
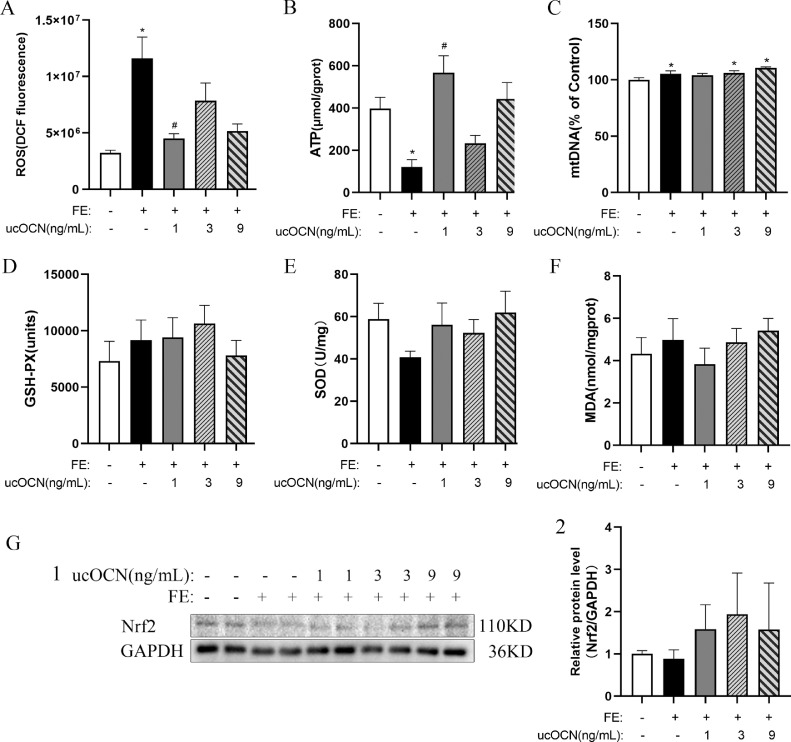
Effects of ucOCN and FE on oxidative stress in chicken embryonic hepatocytes. A, The changes of reactive oxygen species concentrations. B, The changes of adenosine triphosphate concentrations. C, The changes of mitochondrion DNA percentage. D, The changes of glutathione peroxidase concentrations. E) The changes of superoxide dismutase concentrations. F, The changes of malondialdehyde concentrations. G1-2, The change of Nrf2 relative protein expression. The data represent Mean ± SEM (n = 6 per group). Differences were determined by one-way ANOVA followed by LSD test. *P < 0.05 compared with the control group, ^#^*P* < 0.05 compared with the FE group. Abbreviations: OCN, osteocalcin; FE, fat emulsion; ROS, reactive oxygen species; ATP, adenosine triphosphate; mtDNA, mitochondrion DNA; GSH-Px, glutathione peroxidase; SOD, superoxide dismutase; MDA, malondialdehyde; Nrf2, nuclear factor like-2.

**Figure 3:**
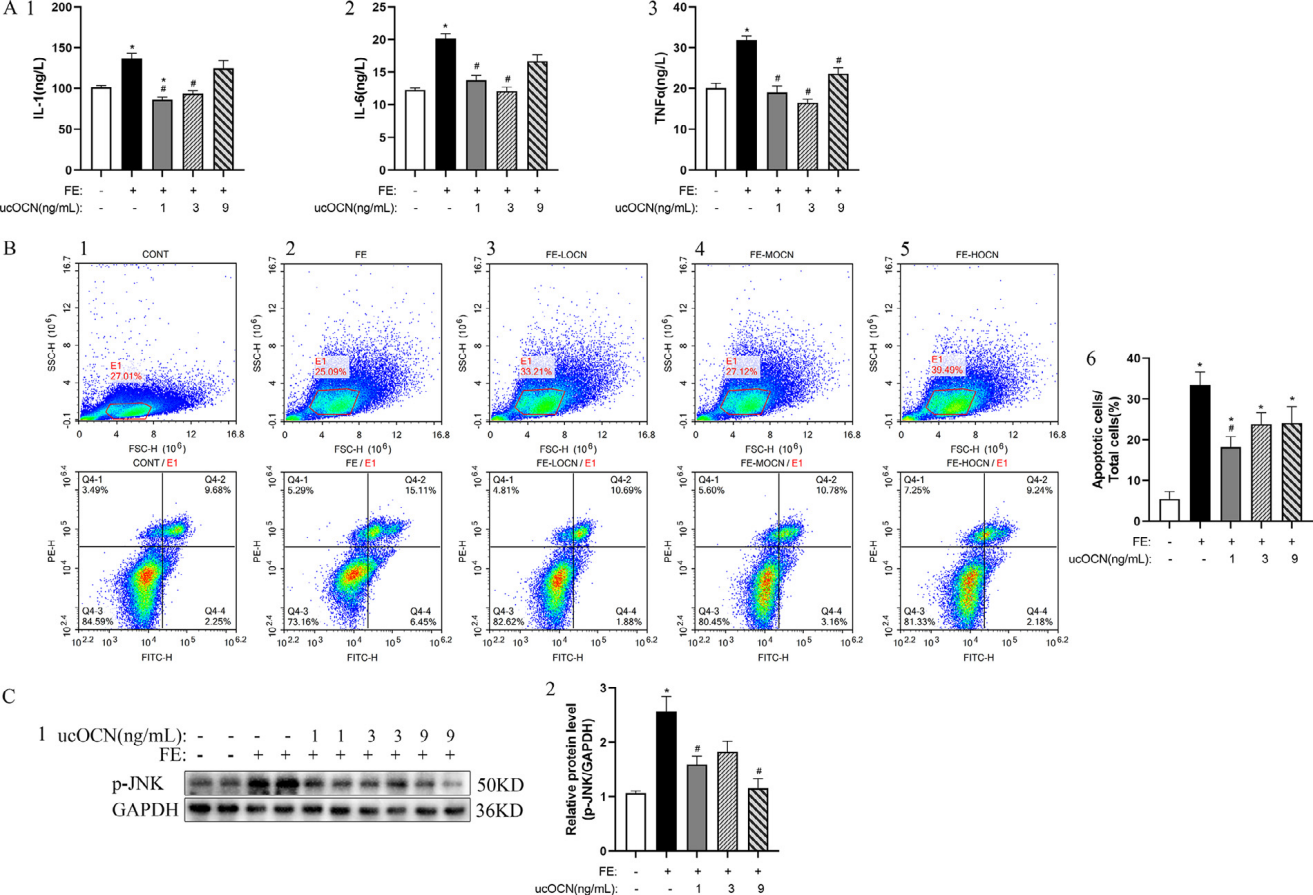
Effects of ucOCN and FE on inflammatory reaction, apoptosis and p-JNK in chicken embryonic hepatocytes. A1-3, The change of IL-1, IL-6 and TNF-α concentrations. B1-6, Apoptotic ratio analyzed by flow cytometry. C1-2, The change of p-JNK relative protein expression. The data represent Mean ± SEM (n = 6 per group). Differences were determined by one-way ANOVA followed by LSD test. *P < 0.05 compared with control group, ^#^*P* < 0.05 compared with FE group. Abbreviations: OCN, osteocalcin; FE, fat emulsion; IL-1, Interleukin-1; IL-6, Interleukin-6; TNF-α, Tumor Necrosis Factor-alpha; p-JNK, phosphorylated c-Jun N-terminal kinase.

Compared with the CONT cells, FE treated hepatocytes had an increased ROS concentration (*P* < 0.05; Figure 2A) and a decreased ATP concentration (*P* < 0.05; Figure 2B). ucOCN administration alleviated these effects, especially, FE-LOCN restrained ROS concentration (*P* < 0.05) and enhanced ATP concentration (*P* < 0.05) compared to the FE group. Compared to the control group, FE (*P* < 0.05; Figure 2C), 3 or 9 ng/mL ucOCN (*P* < 0.05) elevated mtDNA level in hepatocytes. There was no treatment effect on the hepatocyte GSH-Px, SOD, MDA concentrations and Nrf2 protein levels (Figure 2D–G).

### Effects of ucOCN on Inflammatory and Apoptosis in Chicken Embryonic Hepatocytes

Compared with the control group, all inflammatory factors including IL-1 (*P* < 0.05; Figure 3A1), IL-6 (*P* < 0.05; Figure 3A2), and TNF-α (*P* < 0.05; Figure 3A3) were significantly increased in the FE group cells. Both ucOCN at 1 and 3 ng/mL inhibited FE effects on all measured inflammatory factors (*P* < 0.05, respectively), while ucOCN at 9 ng/mL affected FE effects on TNF-a only (*P* < 0.05). Flow cytometry analysis showed that FE increased the intercellular complexity due to the higher SSC-H level (Figure 3B2) and the apoptosis rate (*P* < 0.05; Figure 3B6), while the 1 ng/mL ucOCN could relieved these effect (*P* < 0.05; Figure 3B2, B6). In addition, compared with the FE group, 1 and 9 ng/mL ucOCN significantly suppressed the expression of p-JNK (*P* < 0.05; Figure 3C1-2).

### ucOCN Reduces the Fat Accumulation and Inflammatory Reaction by Inhibiting ROS-JNK Signal Pathway

**Figure 4:**
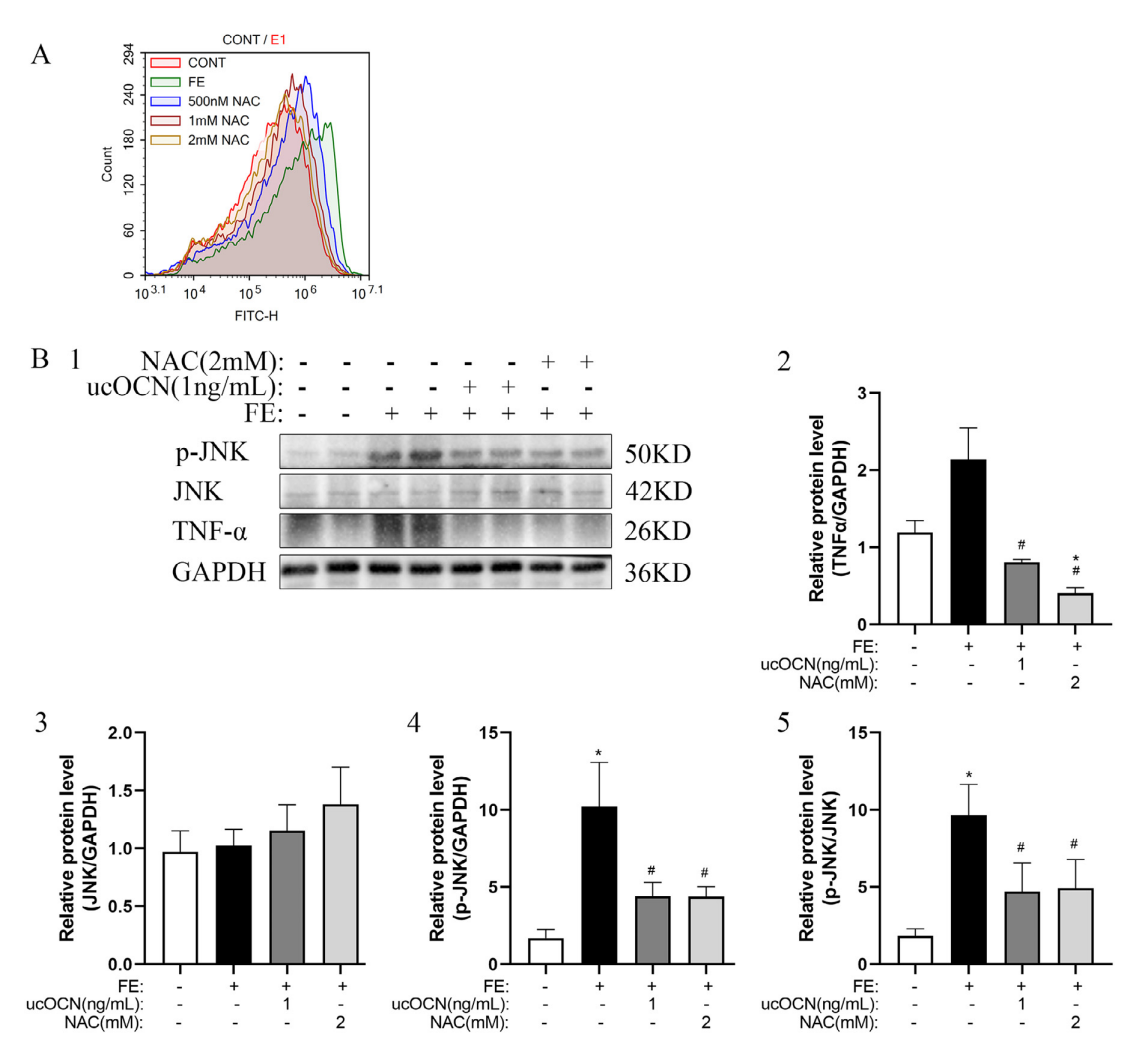
Effects of NAC (a ROS scavenger) on TNF-α and p-JNK/ JNK in chicken embryonic hepatocytes. A, The effects of ROS after NAC (0.5, 1, and 2 mM) treatment. B1-5, The change of TNF-α, JNK, and p-JNK relative protein expression. The data represent Mean ± SEM (n = 6 per group). Differences were determined by one-way ANOVA followed by LSD test. *P < 0.05 compared with the control group, ^#^*P* < 0.05 compared with the FE group. Abbreviations: OCN, osteocalcin; FE, fat emulsion; NAC, N-Acetyl-L-cysteine, ROS scavenger; TNF-α, Tumor Necrosis Factor-alpha; JNK, c-Jun N-terminal kinase.

The performance of NAC (ROS scavenger) at different concentrations was detected by flow cytometry. The results showed that 2 mM NAC could eliminate ROS concentration to the control levels (Figure 4A). The further detection showed that compared with the FE hepatocyte, the 2 mM NAC significantly decreased TNF-α (*P* < 0.05; Figure 4B1-2) and p-JNK protein levels (*P* < 0.05; Figure 4B1, B4) and p-JNK/JNK ratio (*P* = 0.05; Figure 4B5), which was similar as the effects of OCN.

**Figure 5:**
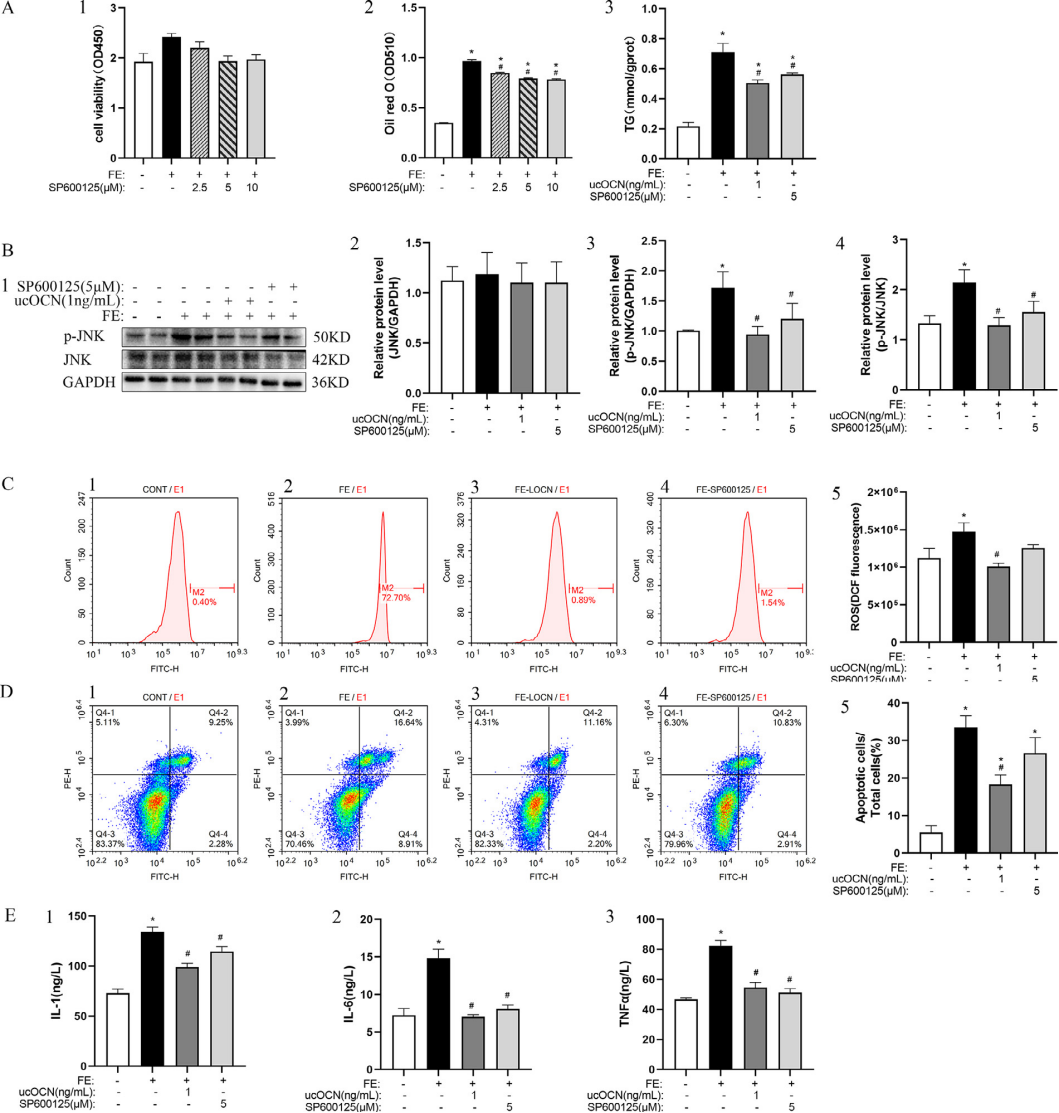
ucOCN reduces the inflammatory reaction and fat accumulation by inhibiting ROS-JNK signal pathway. (A1-3), The cell viability and fat accumulation detected by oil red O and TG. B1-4, The change of JNK and p-JNK relative protein expression. C1-5, The ROS concentrations detected by flow cytometry. D1-5, Apoptotic ratio analyzed by flow cytometry. E1-3, The change of IL-1, IL-6, and TNF-α concentrations. The data represent Mean ± SEM (n = 6 per group). Differences were determined by one-way ANOVA followed by LSD test. *P < 0.05 compared with the control group, ^#^*P* < 0.05 compared with the FE group. Abbreviations: OCN, osteocalcin; FE, fat emulsion; SP600125, JNK inhibitor; TG, triglyceride; JNK, c-Jun N-terminal kinase. ROS, reactive oxygen species; IL-1, Interleukin-1; IL-6, Interleukin-6; TNF-α, Tumor Necrosis Factor-alpha.

Although 2.5, 5, and 10 μM SP600125 (JNK inhibitor) did not influence the hepatocytes viability (Figure 5A1), 5 µM SP600125 significantly inhibited FE caused increase of oil and TG concentrations in hepatocytes (*P* < 0.05; Figure 5A2-3). Based on the results, 5 µM SP600125 was used to following analyses. Western-blotting analysis showed that compared with the FE group, both of 1 ng/mL ucOCN and 5 µm SP600125 significantly reduced p-JNK levels and p-JNK/JNK ratio (Figure 5B1-4). FE increased ROS concentration (*P* < 0.05; Figure 5C1-5) and apoptosis rate (*P* < 0.05; Figure 5D1-5) in the hepatocytes, which was significantly inhibited by ucOCN but not SP600125 Similar to the function of OCN, SP600125 significantly suppressed the increase of IL-1, IL-6 and TNF-α concentrations (*P* < 0.05; Figure 5E1-3) in hepatocytes induced by FE.

## DISCUSSION

Primary chicken hepatocytes can be isolated from freshly perfused chicken liver, but the mixed cells from chicken liver also contain hepatic nonparenchymal cells including macrophages, stellate cells or biliary endothelial cells (Mackei et al., 2020). It is easier to get purified hepatocytes using chicken embryonic livers (Li et al., 2018; Wu et al., 2021a). The purified hepatocytes can be identified by PAS staining which is used to specifically identify them by the markers of carbohydrates and stored glycogens (Hui et al., 2017; Tao et al., 2021). In the current study, FE resulted in a high level of TG and a mass of LDs in hepatocytes, which suggests that FE successfully induces hepatocytic steatosis. In addition, FE significantly increased the hepatocyte viability. It may indicate that FE provides an additional nutritional supply to induce proliferation of hepatocytes (Urso and Zhou, 2021). And ucOCN further promoted the hepatocyte viability, which means ucOCN is advantageous to proliferate hepatocytes. In mice, ucOCN alleviates fatty accumulation in the NAFLD livers (Zhang et al., 2021) and decreases the TG concentration in the hepatocytes (Xia et al., 2021; Zhang et al., 2021). Similarly, ucOCN decreased TG concentration and LDs number in hepatocytes, suggests that ucOCN inhibits hepatocytic steatosis. Moreover, Zhang et al. (2020) reported that ucOCN had a dose-dependent (0, 3, and 30 ng/mL) attenuation of mice hepatocytic steatosis (Zhang et al., 2020). However, in our study, 1 ng/mL was the optimal concentration of ucOCN for improving hepatocyte viability and reducing fat accumulation, which may clue that chicken embryonic hepatocytes may be more sensitive to ucOCN than that of mammals.

Liver biopsies of NASH patients and NAFLD mice show mitochondria function defection in hepatocytes (Pirola et al., 2013; Einer, et al., 2018). Accumulated fatty acids induce harmful ROS with lipotoxicity which occurs as a process of altered mitochondrial oxidative metabolism in rat hepatocytes (Egnatchik et al., 2014). Mitochondrial dysfunction leads to low ATP and high mtDNA (Chistiakov et al., 2018; Singh et al., 2019; Wang et al., 2019). For example, ATP was depleted in the liver of fructose-induced NAFLD mice (Softic et al., 2016), and mtDNA is released into the cytosol in palmitic acid-induced primary mouse hepatocytes (Gao et al., 2020). In the current study, the transmission electron microscopic images displayed that FE caused great mitochondrial swelling with membranolytic, cracked or decreased of crista without effects on the mitochondria number, which indicates FE leads to the damage of mitochondrial structure. FE increased the ROS concentration, decreased the ATP concentration, and raised the copy number of mtDNA, which implies that FE-induced excessive fat accumulation produces lipotoxicity with damaged mitochondrial function. The loss of mitochondrial integrity may cause dysfunction of cell membrane sodium and potassium pump, by which it further leads to excessive accumulation of sodium ions and water within the cells, inducing hepatocyte edema (Hasan et al., 2018; Garner et al., 2021). Yilmaz et al. (2011) reported that patients with biopsy proven NAFLD had significant reduction in serum ucOCN concentrations compared with that of health people, which was significantly associated with the extent of hepatocyte ballooning. The current study displayed that 1 ng/mL ucOCN significantly reduced the ROS concentrations and mtDNA copy number, increased the ATP concentration, alleviated the mitochondrial swelling and hepatocytes edema, which may indicate that ucOCN improves mitochondrial dysfunction. Mitochondrial dysfunction is closely related to oxidative stress (Egnatchik et al., 2014). However, FE and ucOCN had no significant effects on MDA concentration, Nrf2 protein level, and antioxidant enzyme (SOD and GSH-Px) activity in the current study. It is possible that mitochondrial damage can lead to hepatocytes edema without significant effects on oxidative stress. Therefore, ucOCN can play a key role in maintaining mitochondrial structural and functional homeostasis.

In a vitro study, FE and ucOCN have opponent functions in regulating p-JNK protein expression in rat hepatocytes (Egnatchik et al., 2014). EF enhances while ucOCN alleviates p-JNK protein expression, which may indicate that FE activates JNK signaling pathway and ucOCN inhibits thepathway. Excessive fatty acids produce harmful ROS with lipotoxicity in rat hepatocytes (Egnatchik et al., 2014). FE and CON also show the opponent function in ROS production. FE increases while ucOCN reduces the ROS concentration in chicken embryonic hepatocytes, suggesting that ucOCN can suppress the ROS damage in steatosis hepatocytes. The current study showed that NAC, a ROS scavenger, had the similar function as ucOCN that not only clears ROS but also decreases p-JNK protein expression, which further supports the previous findings that ROS directly stimulates JNK pathway (Sun, et al., 2019; Suzuki et al., 2015). SP600125, a JNK inhibitor, without effects on ROS production in FE-induced hepatocytes, may clue that JNK may regulate ROS with negative feedback. Therefore, ucOCN can regulate hepatocytic functions via inhibiting ROS-JNK signal pathway.

Inhibition of JNK reduces steatosis and steatohepatitis of the liver in HFD-induced rats (Yan et al., 2017), and decreases fat accumulation in human HepG2 hepatoma cells via the ROS/JNK/AP-1 signaling (Xie et al., 2021). Similar to the outcomes of these studies, both SP600125 and ucOCN alleviated FE-induced high TG concentration in the current study, indicating that ucOCN suppresses fat accumulation by ROS-JNK signaling in chicken embryonic hepatocytes.

Our previous study had showed that the HFD promoted the FLHS development charactered by increased liver hemorrhage score and fibrosis in aged laying hens (Wu et al., 2021b). In a current parallel study, it further showed the HFD enhanced the liver TNF-α concentrations, indicating the HFD-induced FLHS has severe inflammatory reactions (Unpublished data). Meantime, these studies showed that ucOCN reduced the gene and protein expressions of TNF-α in the liver, suggesting that ucOCN inhibits the occurrence of steatohepatitis in hens. These results are consistent with mammal NAFLD. In obese mice, ucOCN reduces the expression of inflammation and inflammasome related genes, including TNF, IL-1β, IL-6, and NLRP3 (Guedes et al., 2018). FE-induced inflammatory reactions increased p-JNK/JNK protein expression and inflammatory cytokine including IL-1, IL-6, and TNF-α concentrations can be blocked by SP600125 and OCN, proving that ucOCN reduces inflammation via ROS-JNK signal pathway in hepatocytes.

**Figure 6:**
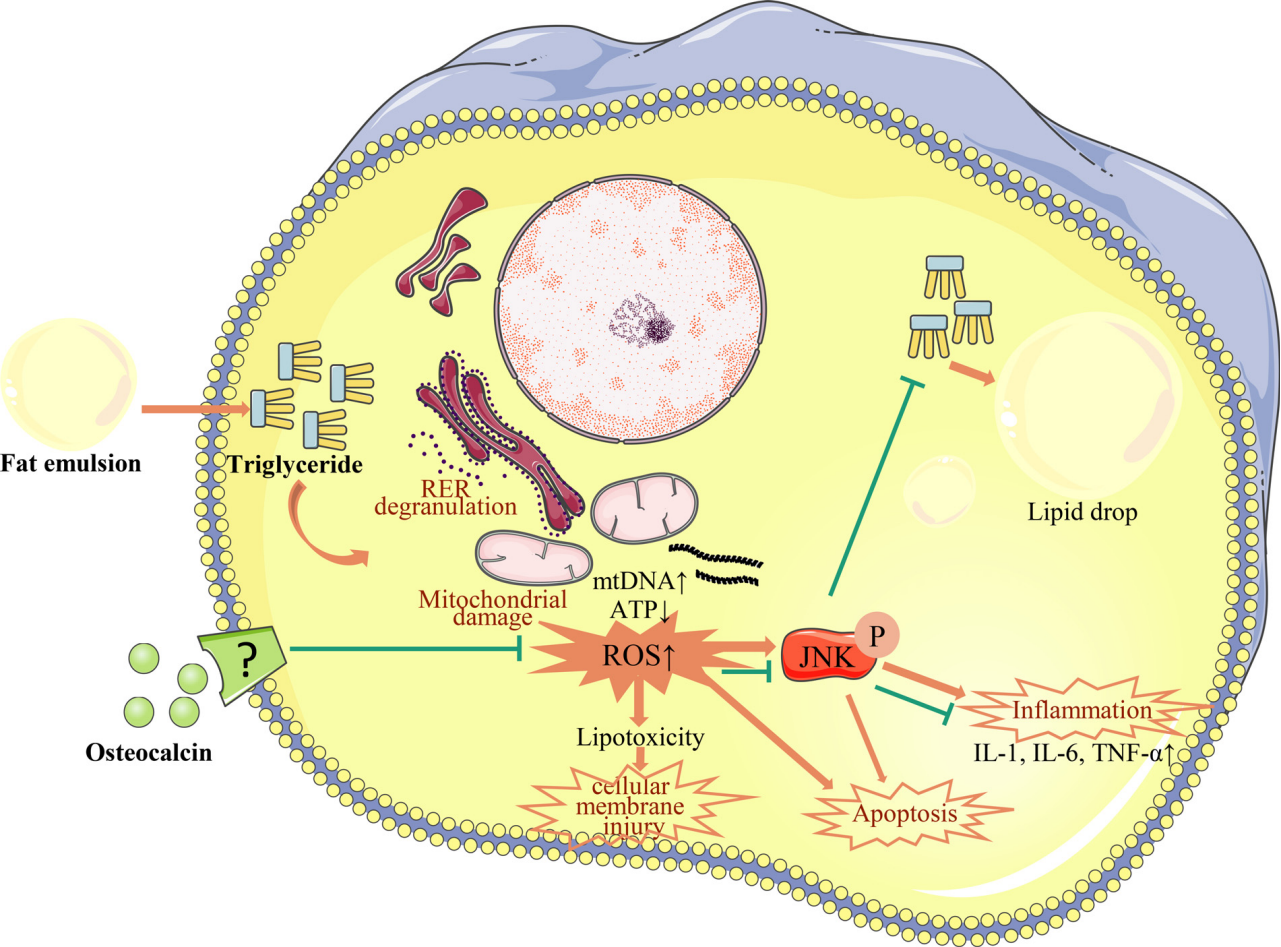
Effects of osteocalcin on fat emulsion-induced fat accumulation and inflammatory reactions in chicken embryonic hepatocytes.

Several studies have shown that the activation of ROS-JNK signaling pathway promotes apoptosis (Li et al., 2020c; Wang et al., 2020) and JNK is an activator of apoptosis (Lu et al., 2021). FE increased hepatocytes apoptosis rate, which can be contributed to the activation of ROS-JNK (Chang et al., 2006; Wang et al., 2014). In the current stuyd, low-dose (1 ng/mL) but not high-dose (9 ng/mL) of ucOCN inhibited apoptosis in hepatocytes It indictes that 1 ng/mL ucOCN is the suitable concentraton for regulating hepatocytes. The result is also consistent with the 1 ng/mL ucOCN improving hepatocyte viability and reducing fat accumulation. ucOCN but not SP600125 has significantly relieved the effect of FE on hepatocytes, suppressing hepatocyte apoptosis via both ROS-JNK signal pathway as well as other regulating pathways such as lipotoxicity (Kusminski et al., 2009). The studies examining the underlying mechanisms of ucOCN effects on preventing hepatocytic damage are ongoing (Figure 6).

## CONCLUSION

The results of this study suggest that fat emulsion promotes lipid droplets accumulation, mitochondrial damage, cellular edema, inflammatory reaction, and apoptosis in primary chicken embryonic hepatocytes. Osteocalcin functionally alleviates hepatocytes damage, mitochondrial damage, ROS concentration, fat accumulation, inflammatory cytokine production and apoptosis via inhibiting ROS-JNK signaling pathway.
